# [^18^F]FSPG-PET reveals increased cystine/glutamate antiporter (xc-) activity in a mouse model of multiple sclerosis

**DOI:** 10.1186/s12974-018-1080-1

**Published:** 2018-02-22

**Authors:** Aileen Hoehne, Michelle L. James, Israt S. Alam, John A. Ronald, Bernadette Schneider, Aloma D’Souza, Timothy H. Witney, Lauren E. Andrews, Haley C. Cropper, Deepak Behera, Gayatri Gowrishankar, Zhaoqing Ding, Tony Wyss-Coray, Frederick T. Chin, Sandip Biswal, Sanjiv S. Gambhir

**Affiliations:** 1 Molecular Imaging Program at Stanford (MIPS), Department of Radiology, Stanford University, Stanford, CA 94305 USA; 2 Department of Neurology and Neurological Sciences, Stanford University, Stanford, CA 94305 USA; 3 Department of Bioengineering, Department of Materials Science & Engineering, Stanford University, Stanford, CA 94305 USA

**Keywords:** FSPG, PET, Multiple sclerosis, EAE mice, Xc-

## Abstract

**Background:**

The cystine/glutamate antiporter (xc-) has been implicated in several neurological disorders and, specifically, in multiple sclerosis (MS) as a mediator of glutamate excitotoxicity and proinflammatory immune responses. We aimed to evaluate an xc-specific positron emission tomography (PET) radiotracer, (4*S*)-4-(3-[^18^F]fluoropropyl)-l-glutamate ([^18^F]FSPG), for its ability to allow non-invasive monitoring of xc- activity in a mouse model of MS.

**Methods:**

Experimental autoimmune encephalomyelitis (EAE) was induced in C57BL/6 mice by subcutaneous injection of myelin oligodendrocyte glycoprotein (MOG_35–55_) peptide in complete Freund’s adjuvant (CFA) followed by pertussis toxin. Control mice received CFA emulsion and pertussis toxin without MOG peptide, while a separate cohort of naïve mice received no treatment. PET studies were performed to investigate the kinetics and distribution of [^18^F]FSPG in naïve, control, pre-symptomatic, and symptomatic EAE mice, compared to ^18^F-fluorodeoxyglucose ([^18^F]FDG). After final PET scans, each mouse was perfused and radioactivity in dissected tissues was measured using a gamma counter. Central nervous system (CNS) tissues were further analyzed using ex vivo autoradiography or western blot. [^18^F]FSPG uptake in human monocytes, and T cells pre- and post-activation was investigated in vitro.

**Results:**

[^18^F]FSPG was found to be more sensitive than [^18^F]FDG at detecting pathological changes in the spinal cord and brain of EAE mice. Even before clinical signs of disease, a small but significant increase in [^18^F]FSPG signal was observed in the spinal cord of EAE mice compared to controls. This increase in PET signal became more pronounced in symptomatic EAE mice and was confirmed by ex vivo biodistribution and autoradiography. Likewise, in the brain of symptomatic EAE mice, [^18^F]FSPG uptake was significantly higher than controls, with the largest changes observed in the cerebellum. Western blot analyses of CNS tissues revealed a significant correlation between light chain of xc- (xCT) protein levels, the subunit of xc- credited with its transporter activity, and [^18^F]FSPG-PET signal. In vitro [^18^F]FSPG uptake studies suggest that both activated monocytes and T cells contribute to the observed in vivo PET signal.

**Conclusion:**

These data highlight the promise of [^18^F]FSPG-PET as a technique to provide insights into neuroimmune interactions in MS and the in vivo role of xc- in the development and progression of this disease, thus warranting further investigation.

**Electronic supplementary material:**

The online version of this article (10.1186/s12974-018-1080-1) contains supplementary material, which is available to authorized users.

## Background

The key pathological hallmarks of multiple sclerosis (MS) include demyelination, oligodendrocyte cell death, microglial activation, peripheral immune cell infiltration, and axonal degeneration in the central nervous system (CNS) [1]. Over the past two decades, an increasing amount of data has emerged suggesting that oxidative stress also plays a key role in the pathogenesis of MS [2]. It is known that upregulation of cystine/glutamate antiporter (xc-) is an oxidative stress response by cells in an attempt to increase availability of the glutathione biosynthesis precursor cystine for improved reactive oxygen species (ROS) detoxification [3]. Under certain pathological conditions accompanied by dysregulation of glutamate homeostasis, increased cystine influx through xc- leads to elevated extracellular levels of the neurotransmitter glutamate [4]. Glutamate excitotoxicity is known to contribute to oligodendrocyte and tissue damage in MS by over-activating glutamate receptors [5]. In 2011, Pampliega and colleagues reported that the light chain of the heterodimeric xc- transmembrane protein (known as xCT) responsible for its transporter activity is upregulated in the spinal cord of mice and rats with experimental autoimmune encephalomyelitis (EAE), the most widely characterized rodent model of MS, as well as human MS postmortem tissue [6]. A recent study by Merckx and colleagues suggests that it is the peripheral immune cells expressing xc-, known to infiltrate the CNS in MS, rather than the resident immune cells that directly contribute to glutamate release and excitotoxicity [7].

Recently, Martin and colleagues described the use of a previously reported ^18^F-labeled small molecule xc- substrate—known as (4S)-4-(3-[^18^F]fluoropropyl)-l-glutamate ([^18^F]FSPG)—for imaging xc- activity in EAE rats using PET [8]. While these studies highlighted the promise of [^18^F]FSPG-PET imaging as a non-invasive biomarker of MS disease status and for therapy monitoring, the authors did not investigate the relationship between the observed increased PET signal in EAE rats and levels of target expression. Here, we propose to expand upon our previous work with [^18^F]FSPG in MS rodent models [9] by conducting the first investigation of correlation between [^18^F]FSPG-PET signal and levels of xCT in EAE, alongside human immune cell uptake studies, with the aim of improving understanding of the molecular and cellular significance of [^18^F]FSPG signal in EAE and MS. Since the Lewis rat model of EAE does not undergo demyelination [10] (a hallmark feature of human MS), we conducted [^18^F]FSPG-PET imaging in an EAE mouse model that does undergo demyelination, with the aim of obtaining clinically relevant and translatable information concerning this tracer’s potential use in MS.

The primary goal of the present studies was therefore to evaluate [^18^F]FSPG for its ability to detect alterations in xc- antiporter activity in EAE mice at different stages of disease via PET imaging and to correlate PET findings with xCT protein levels in affected CNS tissues.

## Methods

### Study design

Since there were no prior reports of [^18^F]FSPG-PET imaging in EAE mice, we began by performing pilot dynamic PET/CT imaging studies in symptomatic EAE (score 1.0–3.5) versus naive mice to gain an understanding of the in vivo distribution and kinetics of [^18^F]FSPG. Next, we compared the uptake pattern of [^18^F]FSPG with that of the widely used clinical PET tracer, ^18^F-fluorodeoxyglucose ([^18^F]FDG), previously shown to have increased uptake in activated immune cells and/or CNS lesions in EAE rodent models and MS patients [11, 12]. The utility of [^18^F]FDG-PET in MS is limited due to the high/fluctuating basal uptake of this radiotracer in CNS tissues of MS patients, which generates ambiguity in the detection of [^18^F]FDG accumulation in lesions [13, 14]; hence, the need to assess whether [^18^F]FSPG uptake could serve as a more sensitive marker of pathological changes in CNS tissues of EAE mice.

Following these initial comparison studies, we performed longitudinal experiments to characterize [^18^F]FSPG uptake in CNS tissues at the pre-symptomatic versus symptomatic stages of EAE disease and to examine the relationship between imaging signal and xCT protein levels. Lastly, as a prelude to future clinical studies with [^18^F]FSPG, we investigated the uptake of [^18^F]FSPG in activated human monocytes and T cells—which have both been implicated in the pathogenesis of EAE/MS. We chose to use human cells for these studies to add a translational dimension to our work with the goal of obtaining insights into the extent that specific immune cell types contribute to [^18^F]FSPG-PET signal in MS.

### Radiochemistry

[^18^F]FSPG was synthesized according to previous methods [15] in 45–70 min with a radiochemical yield of 36 ± 17% (decay-corrected to end of bombardment), specific activity 48.47 ± 16.65 GBq/μmol (end of synthesis) and radiochemical purity > 90% (*n* = 13). Clinical grade [^18^F]FDG was supplied by the Radiochemistry Facility at Stanford University.

### EAE immunization

Animal procedures were approved by Stanford University Institutional Animal Care and Use Committee. Female C57BL/6 mice (10–12 weeks old) were immunized subcutaneously with 100 μg of myelin oligodendrocyte glycoprotein fragment_35–55_ (MOG_35–55_) peptide (Stanford Protein and Nucleic Acid Biotechnology Facility) emulsified in complete Freund’s adjuvant (CFA; 200 μg mycobacterium tuberculosis, BD Difco Adjuvants). Mice received an intravenous injection of 100 ng pertussis toxin (List Biological Laboratories Inc.) at the time of immunization and 48 h later. Control mice received a CFA/PBS emulsion without MOG peptide and the same amount of pertussis toxin. Mice were weighed, examined daily for clinical signs of EAE, and scored according to the following criteria: score of 0, no paralysis; 0.5, partial loss of tail tone; 1, complete loss of tail tone; 1.5, complete tail paralysis accompanied by weakness in one hind limb; 2, hind limb weakness or paresis; 2.5, paralysis of at least one hind limb or no weight bearing on hind limbs (dragging) but with some leg movement; 3, complete hind limb paralysis; 3.5, hind limb paralysis, unable to right itself when placed on its side; 4, hind limb paralysis and forelimb paresis; 4.5, moribund; and 5, dead. Mice were euthanized at the end of the day once they reached a score of 4.0 and immediately after reaching 4.5.

### PET imaging studies

Naïve, control, and EAE mice were administered [^18^F]FSPG (~ 9.25 MBq) or [^18^F]FDG (~ 4.63 MBq) via tail vein injection. Mice receiving [^18^F]FDG were fasted 5–6 h prior to radiotracer administration and were all given an equal volume of saline i.p. to prevent dehydration. PET imaging of anesthetized mice (1–2% isoflurane) was performed using a microPET/CT hybrid scanner (Inveon, Siemens). All PET images were reconstructed using 2 iterations of three-dimensional ordered subset expectation maximization algorithm (12 subsets) and 18 iterations of the accelerated version of 3D-MAP (i.e., FASTMAP), with a matrix size of 128 × 128 × 159. Attenuation correction was applied to datasets from CT image acquired just prior to each PET scan. No partial volume corrections were performed. Dynamic PET scans were acquired in list mode format over 90 min, starting just prior to radiotracer injection. The resulting data was sorted into 0.5-mm sinogram bins and 19 time frames for image reconstruction (4 × 15 s, 4 × 60 s, 11 × 300 s). For subsequent studies, static PET scans (10 min) were acquired at ~ 70 min after injection of the radiotracer.

Image files were co-registered and analyzed with VivoQuant (version 2.0, inviCRO) (Additional file 1: Figure S1a–c). For VivoQuant analyses, the spine from each mouse was isolated by use of the Otsu thresholding technique and the obtained region of interest (ROI) made immutable. An ROI of the spinal cord was then drawn using the segmented spine as a guide. Corresponding PET data were collected for the whole spinal cord ROI, which was drawn from the caudal end of the skull to the pelvis. For analysis of regional variations in PET signal, the spinal cord was divided, using L1–L5 as landmarks, into two cervical/thoracic and lumbar ROIs, for which separate data were collected. For analysis of the brain, a three-dimensional mouse brain atlas was fitted to the PET/CT images and radioactivity concentrations were obtained using ROIs for the major brain regions (Additional file 1: Figure S1d). From this data, the percentage of injected dose per gram (%ID/g) was calculated for the spinal cord and atlas-based brain regions.

### Tissue collection for ex vivo biodistribution, autoradiography, and Western assays

While still under anesthesia, blood samples (200–400 μL) were collected via cardiac puncture immediately prior to perfusing mice with PBS. After mice had been euthanized, tissues of interest were quickly removed at 110 min post-injection of tracer.

For ex vivo biodistribution assays, tissues were placed in tubes for gamma counting and weighed. Tissue-associated radioactivity was assessed via an automated gamma counter (Cobra II Auto-Gamma counter; Packard Biosciences Co.) and decay-corrected to time of tracer injection using diluted aliquots of the initial administered dose as standards.

A subset of the spinal cords and brains were analyzed via ex vivo digital autoradiography (*n* = 1 for EAE, score 3.0–3.5 and *n* = 1 for naïve mice per tracer). Following perfusion, the brain was removed and embedded in optimal cutting temperature (O.C.T.) compound (Tissue-Tek, Sakura, USA). Subsequently, 20-μm-thick sagittal brain sections were cut using a cryostat microtome HM500 (Microm, Germany). The sections were mounted on microscope slides (Fisherbrand Superfrost™ Plus Microscope Slides), air-dried for 10 min, and then exposed to ^18^F-sensitive storage phosphor screens (Perkin Elmer, USA) for 12 h at 4 °C. The image plates were analyzed using a Typhoon 9410 Variable Mode Imager (Amersham Biosciences, USA), and images were visualized and processed by ImageJ (image processing and analysis software in java, version 1.45s). Anatomy of brain sections was confirmed by staining the exact same sections with Nissl (cresyl violet acetate, Sigma Aldrich). Briefly, dehydrated mounted sections were cleared in xylene for 15 min, rehydrated, immersed in 0.5% cresyl violet acetate (Sigma Aldrich) solution for 10 min, rinsed, dehydrated, and coverslipped. Whole spinal cords were also analyzed via autoradiography using same storage phosphor screens and Typhoon imager.

For Western blotting, the spinal cords were cut into thoracic/cervical and lumbar segments and the right hemisphere of the brain was collected. Tissue samples were immediately frozen in liquid nitrogen and stored at − 80 °C.

### Western blotting

Tissue samples were homogenized using a total protein extraction kit (BioChain) following the manufacturer’s instructions. Protein concentration in lysates was determined by bicinchoninic acid (BCA) protein assay (Pierce). Rabbit anti-xCT antibody (1:2000 dilution, Novus NB300-318) was used according to a standard western blotting protocol. After enhanced chemiluminescence (ECL) exposure, blots were washed and re-probed with rabbit anti-actin antibody (Cell Signaling Technology 4967S; 1:5000) as a loading control. To verify efficient extraction of xCT from the membrane during tissue lysis, an anti-Na,K-ATPase antibody (Cell Signaling Technology 3010S; 1:5000) was used as an additional control for western blotting [16]. Blots were scanned and signal was quantified using ImageJ (National Institutes of Health).

### Isolation and activation of primary human immune cells

Peripheral mononuclear blood cells (PMBCs) were isolated from the whole blood of healthy donors (Stanford Blood Center) via density gradient method (Ficoll Paque PLUS, GE Healthcare). T cells or monocytes were isolated from the PMBC fraction by negative selection (Naïve Pan T Cell Isolation Kit/Monocyte Isolation Kit II, Miltenyi Biotech). Purity of obtained fractions was assessed by staining with CD3-VioBLue and CD14-FITC antibodies (Miltenyi Biotech) for T cells and monocytes respectively. The number of stained cells was then counted as a percentage of total cell number using a fluorescence microscope (EVOS FL Cell Imaging System, Life Technologies).

T cells were maintained in TexMACS medium (Miltenyi Biotech) and an aliquot was activated for 48 h prior to the radiotracer uptake assay using a T Cell Activation/Expansion Kit human (Miltenyi Biotech) according to manufacturer’s instructions. Monocytes were maintained in RPMI medium containing 10% heat-inactivated FBS and 1% penicillin-streptomycin (all from Life Technologies), and an aliquot was also activated for 48 h prior to the uptake assay with lipopolysaccharide (LPS; 100 ng/mL, *Escherichia coli* O11:B4, Sigma Aldrich). Activation was confirmed by assessing changes in morphology of activated versus resting cells.

### Cell uptake studies using primary human immune cells

Cells were counted, washed with Hank’s Balanced Salt solution (HBSS) and re-suspended at 5–10 × 10^6^ cells/mL in HBSS. Five hundred microliters of aliquots of cell suspensions were allowed to recover for 15 min at 37 °C and 5% CO_2_. Five hundred microliters of pre-warmed [^18^F]FSPG solution (1.5 MBq/mL in HBSS) was then added to each tube. After incubation for 60 min at 37 °C and 5% CO_2_, an aliquot of each cell suspension was layered over 200 μL of a 3:1 mixture of silicon oil and mineral oil in a dolphin tube on ice as previously described [17]. Tubes were spun for 3 min at 10,000*g*, and after freezing in liquid nitrogen, the tips containing the cell pellets were cut into gamma counter tubes. Cell-associated radioactivity was assessed using a gamma counter (Cobra II Auto-Gamma counter; Packard Biosciences Co.) and normalized to both the total activity added and cell number.

### Statistical analysis

Statistical analyses were performed using GraphPad (version 6). Data are expressed as mean ± standard deviation, unless otherwise indicated. Two-tailed Mann-Whitney *U* tests were used for group comparisons and a *p* value < 0.05 was considered significant. Two-tailed Wilcoxon matched-pairs signed rank tests were used to analyze repeated measurements in the longitudinal PET study. A Bonferroni-adjusted *p* value of < 0.017 was considered significant when performing multiple comparisons. Correlation analyses were performed using linear regression with goodness of fit designated by *R*^2^ value, 95% confidence levels.

## Results

### [^18^F]FSPG distribution and kinetics in EAE mice using dynamic PET imaging

Time-activity curves obtained from dynamic PET data (Additional file 2: Figure S2) revealed significantly higher [^18^F]FSPG accumulation in the spinal cords of EAE compared to those of naïve mice, from as early as 8 min after tracer administration until the end of scan (*p* = 0.0043, 75 min post-injection), and a strong trend toward higher uptake in the brain (*p* = 0.0571, 75 min post-injection). While [^18^F]FSPG appeared to wash out of tissues from naïve mice over time, it continued to accumulate in the tissues of EAE mice, with a plateau observed in the spinal cords starting at approximately 60 min after injection. An imaging time point at 75 min post-injection was chosen for all subsequent studies to allow for optimal tracer accumulation in target tissues and clearance from non-target tissues.

### [^18^F]FSPG is more sensitive than [^18^F]FDG at detecting molecular changes in the spinal cord and brain of EAE mice

**Fig. 1:**
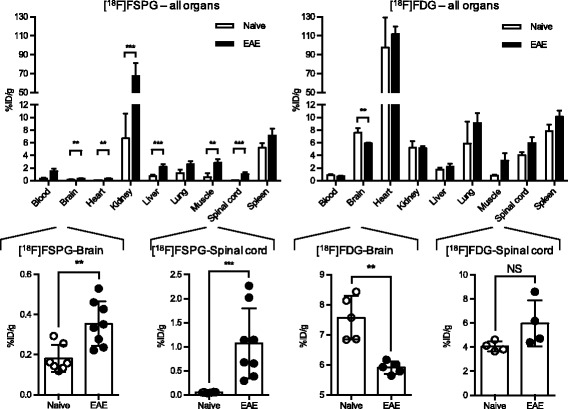
Ex vivo biodistribution of [^18^F]FSPG and [^18^F]FDG in EAE and naive mice. Ex vivo data was obtained 110 min after radiotracer injection (*n* = 5–9, mean %ID/g ± SD) with magnified views of uptake in brain and spinal cord. EAE scores: 2.6 ± 0.9 and 3.2 ± 0.3 for [^18^F]FSPG and [^18^F]FDG groups respectively. ***p 0.0001–0.001, **p 0.001–0.01

Results from ex vivo biodistribution studies demonstrated a significant elevation in [^18^F]FSPG uptake in most EAE tissues compared to that in naïve mice (Fig. 1). The greatest difference in [^18^F]FSPG tissue uptake was seen in the spinal cord, with 1.07 ± 0.70 %ID/g in EAE (*n* = 9, score 2.6 ± 0.9) and 0.05 ± 0.01 %ID/g in naïve mice (*n* = 7), *p* = 0.0007. [^18^F]FSPG uptake was significantly higher in the kidney and brain of EAE compared to naïve mice (kidney: 67.8 ± 40.2 %ID/g vs. 6.7 ± 3.9 %ID/g, *p* = 0.0002; brain: 0.36 ± 0.11 %ID/g vs. 0.18 ± 0.07 %ID/g, *p* = 0.0059). In contrast, there was no significant difference in [^18^F]FDG uptake in most EAE tissues compared to naïve mice (Fig. 1). The only significant difference in [^18^F]FDG uptake was observed in the brain whereby there was a lower accumulation in EAE mice (5.9 ± 0.2 %ID/g) compared to the brains of naïve mice (7.6 ± 0.7 %ID/g, *p* = 0.0079).

Ex vivo autoradiography images corroborated PET and biodistribution findings, illustrating elevated [^18^F]FSPG uptake in the spinal cords of EAE versus naïve mice (Additional file 3: Figure S3A), and a moderate increase in [^18^F]FDG uptake in distinct foci of EAE mice (Additional file 3: Figure S3B). Qualitative autoradiography of selected brains revealed visually higher [^18^F]FSPG uptake in the cerebellum, hippocampus (especially in the dentate gyrus), midbrain reticular nucleus, optic chiasm, posterior complex of the thalamus, and sensory-related substructures of the medulla of EAE compared to naïve mice. In contrast, there was lower global [^18^F]FDG brain uptake in EAE versus naïve mice, most notably in the cortex and olfactory bulb.

### In vivo [^18^F]FSPG-PET imaging reveals increased xc- activity in CNS tissues of EAE mice

Next, we performed a longitudinal study to characterize [^18^F]FSPG uptake at the pre-symptomatic versus symptomatic stage of disease. Since our pilot studies highlighted a tenfold increase in [^18^F]FSPG uptake in EAE mouse kidneys, alongside the increased uptake in the spinal cord and brain, it appeared that [^18^F]FSPG clearance might be affected in EAE mice. To address this, we generated an additional group of control mice with an identical immunization procedure to EAE mice but without administration of MOG peptide antigen. Additionally, we anesthetized mice just prior to PET imaging to allow additional time for clearance of the radiotracer.

**Fig. 2:**
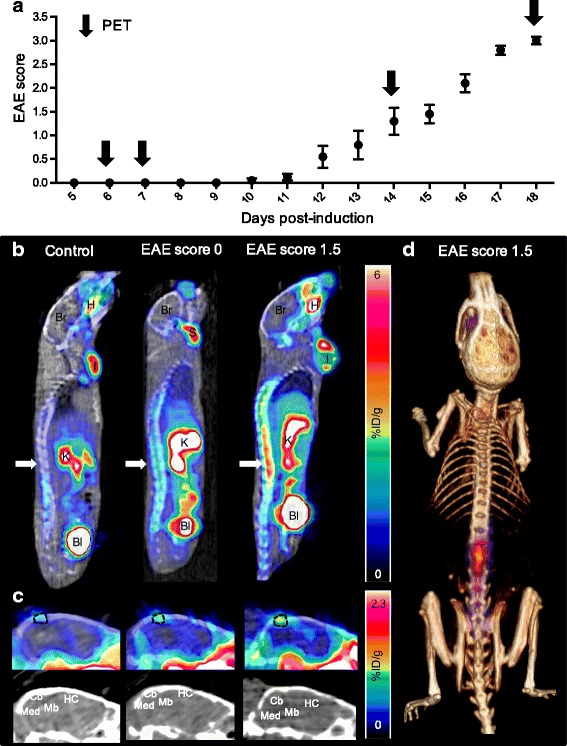
Elevated [^18^F]FSPG-PET signal in EAE mice at early stages of disease. **a** Disease scores of mice induced with EAE over 18 days. Arrows indicate PET/CT imaging time points and scores are represented as mean score ± SEM. **b** Representative sagittal PET/CT images of control, pre-symptomatic EAE (score 0), and EAE mice with early stage disease symptoms (score 1.5). Arrows indicate spinal cord uptake. Bl—bladder, Br—brain, H—Haderian glands, I—immunization site, K—kidneys, S—salivary glands. **c** Representative sagittal PET/CT images of identical brain sections of control, pre-symptomatic EAE (score 0), and symptomatic EAE (score 1.5) mice. Cb—cerebellum, HC—hippocampus, Mb—midbrain, Med—medulla. Dotted lines highlight region containing possible meningeal vessel(s). **d** Representative PET/CT volume rendering technique (VRT) image of EAE mouse (score 1.5). PET signal outside spinal column and skull was removed for better visualization of [^18^F]FSPG uptake in areas of interest

For longitudinal studies, mice underwent an initial static PET/CT scan (~ 70 min post-injection of [^18^F]FSPG) at the pre-symptomatic stage of disease on day 6 or 7 following immunization (Fig. 2a shows disease time course and PET imaging time points). Subsequent PET/CT imaging and biodistribution was performed between days 14 and 18 post-immunization for control and EAE mice after the latter reached a clinical score of 1.0–3.5.

**Fig. 3:**
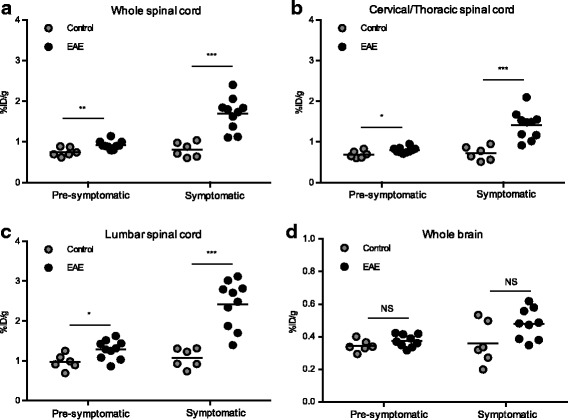
Quantification of [^18^F]FSPG-PET signal in CNS tissues of pre-symptomatic and symptomatic EAE versus control mice. Static PET imaging was performed 75 min post-injection of radiotracer (*n* = 6–10) and signal was quantified in the **a** whole spinal cord, **b** cervical/thoracic spinal cord, **c** lumbar spinal cord, and **d** whole brain. ***p 0.0001–0.001, **p 0.001–0.01, *p 0.01–0.05, NS—non-significant

Longitudinal studies revealed a significant increase in [^18^F]FSPG-PET signal in the spinal cords of EAE mice exhibiting no clinical symptoms (score 0, 1 week post-immunization—Figs. 2 and 3; 1.2 ± 0.1-fold increase, *p* = 0.0047) that later went on to develop pronounced EAE within the next 4–8 days. After the onset of disease, an even greater increase in [^18^F]FSPG uptake was observed in the whole spinal cord of EAE mice compared to that of controls (2.1 ± 0.2-fold higher, *p* = 0.0002). Higher absolute [^18^F]FSPG uptake in the lumbar versus cervical/thoracic spinal cord region was observed (Figs. 2 and 3), while the fold-changes were in the same range (i.e., 1.2 ± 0.1 [*p* = 0.0225] and 1.3 ± 0.1 [*p* = 0.0310] in week 1 and 2.0 ± 0.2 [*p* = 0.0005] and 2.3 ± 0.3 [*p* = 0.0002] in week 2, for cervical/thoracic and lumbar spinal cord, respectively). Although a trend toward higher [^18^F]FSPG uptake in the spinal cord of mice with lower clinical scores was observed, this was not statistically significant. The post-symptomatic signal in the whole spinal cord of EAE mice was 1.8 ± 0.2-fold higher than the pre-symptomatic signal (*p* = 0.0002), and the signal in control mice did not change over time (*p* = 0.5625).

PET image analysis of brain regions revealed significantly elevated uptake in the cerebellum, hippocampus, medulla, and midbrain of symptomatic EAE versus control mice—with the most marked fold-changes in the cerebellum (Table 1). Interestingly, there was a region of radiotracer uptake present in the PET images of EAE mice, which appeared to be just outside the brain (dotted lines in Fig. 2c, see Additional file 4: Figure S4 for coronal brain slices) and became more intense when mice became symptomatic.

**Table 1:** Uptake values (%ID/g) for different brain regions in pre-symptomatic and symptomatic mice and fold differences with respect to the same quantitation in control mice

Brain regions	1 week post-immunization (pre-symptomatic mice)		2 weeks post-immunization (mice with clinical signs of EAE)	
	%ID/g in EAE mice	Fold-differences EAE vs. controls	%ID/g in EAE mice	Fold-differences EAE vs. controls
Whole brain	0.38 ± 0.04	1.1 ± 0.1^NS^	0.48 ± 0.09	1.3 ± 0.2^NS^
Cerebellum	0.36 ± 0.06	1.1 ± 0.1^NS^	0.45 ± 0.10	1.6 ± 0.2^**^
Cortex	0.30 ± 0.04	1.1 ± 0.1^NS^	0.34 ± 0.08	1.2 ± 0.2^NS^
Hippocampus	0.32 ± 0.04	1.1 ± 0.1^NS^	0.38 ± 0.09	1.4 ± 0.2^*^
Hypothalamus	0.76 ± 0.13	1.2 ± 0.1^*^	0.97 ± 0.19	1.2 ± 0.2^NS^
Medulla	0.54 ± 0.08	1.1 ± 0.1^NS^	0.67 ± 0.17	1.5 ± 0.2^*^
Midbrain	0.29 ± 0.04	1.1 ± 0.1^NS^	0.35 ± 0.10	1.5 ± 0.2^*^
Olfactory bulb	0.64 ± 0.15	1.1 ± 0.1^NS^	0.97 ± 0.30	1.3 ± 0.3^NS^
Pallidum	0.45 ± 0.07	1.2 ± 0.1^*^	0.61 ± 0.13	1.2 ± 0.3^NS^
Pons	0.44 ± 0.07	1.0 ± 0.1^NS^	0.71 ± 0.25	1.3 ± 0.3^NS^
Striatum	0.34 ± 0.04	1.1 ± 0.1^NS^	0.42 ± 0.09	1.2 ± 0.2^NS^
Thalamus	0.28 ± 0.05	1.1 ± 0.1^NS^	0.32 ± 0.07	1.3 ± 0.2^NS^

*NS* not significant

Absolute [^18^F]FSPG uptake in brain regions as measured by PET, 75 min p.i. of radiotracer in EAE mice (*n* = 6–10, mean %ID/g ± SD) and fold differences ± SEM versus controls with indices indicating significance by Mann-Whitney test: **p = 0.001–0.01, *p = 0.01–0.05

Elevated [^18^F]FSPG-PET signal in EAE CNS tissues compared to control mice was further confirmed by ex vivo gamma counting of tissues from the same mice (Additional file 5: Fig. S5). Spinal cord [^18^F]FSPG uptake was 0.69 ± 0.35 %ID/g (*n* = 9) in EAE compared to 0.04 ± 0.01 %ID/g in control mice (*n* = 8, *p* < 0.0001), with a similar fold-difference (16.2 ± 3.2) to what we previously observed in pilot studies using naïve mice. Brain uptake was also significantly higher in EAE mice compared to controls (0.15 ± 0.05 %ID/g vs. 0.07 ± 0.03 %ID/g, 2.2 ± 0.4-fold, *p* = 0.0012). Other significant differences in [^18^F]FSPG uptake were observed in the blood (EAE: 0.37 ± 0.15 %ID/g vs. control: 0.20 ± 0.02 %ID/g, *p* = 0.0044) and kidneys (EAE: 6.7 ± 2.4 %ID/g vs. control: 3.8 ± 1.2 %ID/g, *p* = 0.0155).

### [^18^F]FSPG-PET signal correlates with elevated xCT protein levels in EAE CNS tissues

**Fig. 4:**
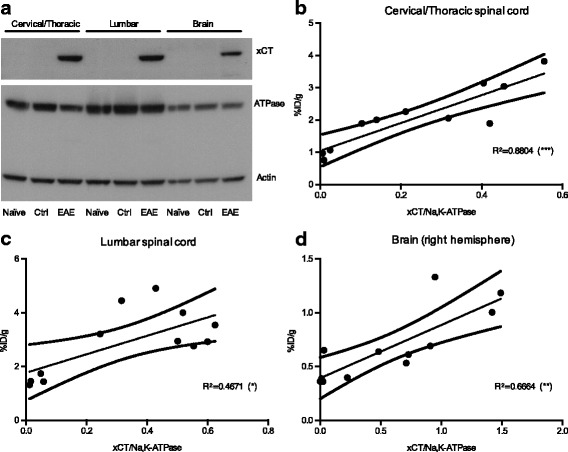
Significant correlation between xCT protein levels in CNS tissues and in vivo PET signal. **a** Representative Western blot with actin and NaK-ATPase as loading controls (Ctrl). Correlation between xCT protein levels (determined by densitometric Western analysis) and [^18^F]FSPG uptake (determined by PET quantification) in the **b** cervical/thoracic spinal cord (SC), **c** lumbar SC, and **d** brain. ***p 0.0001–0.001, **p 0.001–0.01, *p 0.01–0.05

xCT protein levels were assessed by Western blot of the brain, cervical/thoracic, and lumbar cord tissues collected after [^18^F]FSPG**-**PET/CT imaging (Fig. 4 and Additional file 6: Figure S6). Results from these studies revealed a strong correlation between [^18^F]FSPG-PET signal and xCT protein levels in the cervical/thoracic spinal cord (*R*^2^ = 0.80, *p* = 0.0002), and a moderate correlation in the lumbar spinal cord (*R*^2^ = 0.46, *p* = 0.0143) and brain (right hemisphere, *R*^2^ = 0.6664, *p* = 0.0012). xCT expression in the brain and cervical/thoracic and lumbar spinal cords from control versus naïve mice were not significantly different (*p* > 0.05, *n* = 3–4 per group; Additional file 7: Figure S7).

### [^18^F]FSPG uptake is increased in isolated primary human immune cells upon activation

**Fig. 5:**
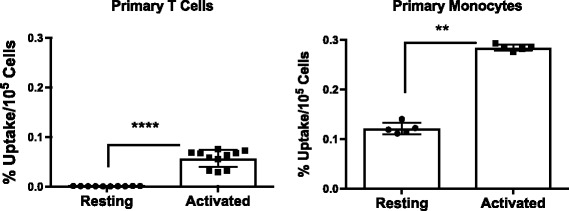
[^18^F]FSPG uptake in resting and activated primary human immune cells. In vitro accumulation of [^18^F]FSPG in isolated primary human T cells and monocytes (*n* = 5–11, mean %ID/g ± SD). ****p < 0.0001, **p < 0.01

Finally, we investigated the uptake of [^18^F]FSPG in primary human T cells and monocytes; the immune cell types which are implicated in the pathogenesis of EAE. Data from these studies revealed a dramatic increase in [^18^F]FSPG uptake in activated compared to resting T cells (75-fold, *p* < 0.0001) and in activated versus resting human monocytes (2.3-fold, *p* = 0.0079, Fig. 5). The absolute level of [^18^F]FSPG uptake in activated monocytes (0.284 ± 0.006%uptake/10^5^ cells), however, was fourfold higher than that in activated T cells (0.070 ± 0.004%uptake/10^5^ cells).

## Discussion

Cystine/glutamate antiporter, xc-, has been implicated in several neurological disorders and, specifically, in MS as a mediator of glutamate excitotoxicity and immune regulation [4]. Recent studies demonstrated that the xCT subunit of system xc- (responsible for the activity of this antiporter) is upregulated in postmortem CNS tissues from both MS patients and MS rodent models [6] and that it might be a promising PET imaging biomarker of disease progression in EAE rats [8].

Here, we show that [^18^F]FSPG-PET enables the sensitive detection of alterations in xCT protein levels/xc- activity in EAE mice at different stages of disease in vivo. Most notably, [^18^F]FSPG-PET showed potential for the early detection of MS due to its increased uptake in the spinal cord of EAE mice even at the pre-symptomatic stage of disease. This is the first report that [^18^F]FSPG-PET signal is elevated at this early stage of EAE, thus emphasizing the utility of [^18^F]FSPG-PET as a technique for characterizing the in vivo role of this antiporter in the early stages of EAE disease development. Previously reported studies performed by Martin and colleagues using [^18^F]FSPG-PET for imaging xc- in EAE rats did not detect increased PET signal at the pre-symptomatic stage of EAE; however, they did not image control or naïve rats for direct comparison [8].

Here, [^18^F]FSPG showed greater sensitivity than [^18^F]FDG in detecting metabolic changes in the EAE spinal cord, exhibiting a 21-fold difference in uptake in EAE vs naïve mice in ex vivo biodistribution studies. [^18^F]FDG biodistribution results from EAE versus naïve and control mice were comparable to those previously reported by Radu et al. [18], Martin et al. [8], and Buck et al. [11]. As in our study, the latter found no statistical significance between groups. The heterogeneous pattern of [^18^F]FDG uptake in distinct inflammatory infiltrates in the spinal cords of EAE mice and significant decrease in [^18^F]FDG uptake in the brain, observed by autoradiography, was also consistent with previous findings [14, 15]. Grassiot and colleagues reported hypometabolism in MS patients and postulated that low [^18^F]FDG brain uptake is likely due to reduced brain volume and disturbed glucose metabolism in these patients [19]. One reason for the difference in sensitivity between [^18^F]FSPG and [^18^F]FDG is likely due to their different uptake mechanisms, and therefore the different biological phenomena these tracers measure. [^18^F]FDG is taken up via glucose transporters and subsequently trapped in cells following phosphorylation by hexokinase II, while [^18^F]FSPG is taken up via xc- and retained in cells by an unconfirmed mechanism [15]. It is possible that alterations in xc- activity are more striking than alterations in glucose metabolism in rodent EAE.

Both PET and autoradiography images indicated higher accumulation of [^18^F]FSPG in the cerebellum, hippocampus, medulla, and midbrain of EAE mice, all regions where demyelinating lesions and tissue damage can occur in MS patients [20] and EAE mice [21]. These findings correspond well with what is known about the spatial distribution of infiltrating immune cells, known to overexpress xCT, in the CNS of EAE rodents. For example, Brown and colleagues reported marked infiltration of CD3-positive T cells and also the presence of activated microglia in the sensory-related substructures of the medulla and cerebellar peduncles of EAE mice [21]. We additionally observed a region with tracer uptake of variable intensity just outside the brain. This signal could possibly be associated with [^18^F]FSPG signal in CD3-positive T cells (or other infiltrating immune cells) in the meninges/subarachnoid space [22] or a meningeal lymphatic vessel [23]—all of which have been shown to be sites where T cells can reside as they mobilize and cross the BBB.

We observed significantly higher [^18^F]FSPG levels in the blood and kidneys of EAE mice compared to naïve mice. The higher kidney uptake is likely due to impaired renal function in EAE mice, a co-morbidity with low prevalence in MS patients [24], leading to inferior excretion ability [25]. The increased level of [^18^F]FSPG in the blood is most likely due to increased circulating activated monocytes in EAE mice, which are known to have elevated xCT protein levels [6]. It is important to note that the measured difference in [^18^F]FSPG uptake levels in CNS tissues of EAE versus naïve/control mice became even more pronounced after removal of the blood via perfusion (as seen in ex vivo biodistribution data) compared to that in in vivo PET data. Hence, it appears that the increased blood pool activity observed in EAE mice partially obscures the specific signal increase. As previously demonstrated by Ayzenberg et al., the effect of EAE immunizations must be considered when interpreting bulk imaging signals by comparing to appropriate controls (i.e., control mice which undergo incomplete immunization with CFA emulsion and pertussis toxin while omitting MOG peptide, rather than using naïve mice) in order to prove specificity of the readout [26]. In this study, we demonstrated specificity of the observed in vivo [^18^F]FSPG-PET signal by using such control mice and also by performing Western blotting of brain and cervical/thoracic and lumbar spinal cord to investigate how well the PET signal reflected alterations in xCT expression. A significant correlation was found between the levels of xCT protein and the PET signal in these tissues, thus verifying the utility of [^18^F]FSPG-PET for investigating in vivo alterations in xCT over time in this mouse model.

As a prelude to clinical evaluation of [^18^F]FSPG in MS patients, we investigated whether a single immune cell population might be primarily responsible for the elevated tissue uptake of [^18^F]FSPG. A recent study by Evonuk and colleagues linked xCT expression on activated T cells with CNS infiltration and disease progression, highlighting the possible utility of xc- as a theranostic target in MS [27]. Numerous studies have highlighted considerable infiltration of CD68-positive cells (macrophages/microglia), monocytes, as well as T cells in spinal cord and brain lesions of EAE rodents [6]. We performed tracer uptake studies in isolated primary human T cells and monocytes to gain clarity on the relative accumulation of [^18^F]FSPG in each cell type. Results from these studies revealed a dramatic 75-fold increase in [^18^F]FSPG uptake in activated versus resting T cells and a comparatively modest, yet significant, 2.3-fold increase in activated versus resting monocytes. These results are in agreement with studies which have shown that T cells only express xCT in the activated state [28]*,* while monocytes constitutively express xCT with upregulation upon activation [6]. CD11b/CD45-positive cells account for the majority of immune cells found in the CNS tissue of EAE mice (40–56% at all stages of disease), whereas cells of the T cell lineage represent a lower fraction (approximately 17%) [29]. Together with the higher absolute tracer uptake in monocytes compared to T cells, it is suspected that both types of immune cells likely contribute to the increased [^18^F]FSPG tissue signal. Moreover, microglia/macrophages likely also contribute to the observed elevated [^18^F]FSPG signal, as [^18^F]FSPG-PET signal in EAE rats was significantly reduced after selectively depleting microglia via intracerebroventricular injection of liposome-encapsulated clodronate [8]. Further characterization of the immune cell populations (both murine and human) predominantly responsible for the increased [^18^F]FSPG tissue uptake at different stages of disease progression are planned and will likely provide insight into the spatiotemporal dynamics of immune cells and their oxidative stress levels in MS.

## Conclusion

In summary, we have shown that [^18^F]FSPG-PET is a promising imaging technique for non-invasively monitoring xc- activity in EAE mice, both at very early stages of disease and throughout progression, and holds great potential as a sensitive tool for investigating the links between oxidative stress, immune cell trafficking, and clinical symptoms of MS. Using this technique, we gained new insights into the in vivo spatiotemporal dynamics of xc- activity in pre-symptomatic and symptomatic EAE. Future studies will involve evaluating the use of [^18^F]FSPG-PET for predicting/monitoring response to novel antioxidant therapeutics, which are gaining interest as neuroprotective agents in MS.

## Additional files

Additional file 1: Methods for defining regions of interest (ROIs) in CNS using VivoQuant image analysis software. (a) Spine/vertebrae (blue) was segmented out using Otsu Thresholding. (b) The spine ROI was made immutable, and the thoracic/cervical (orange) and lumbar (yellow) spinal cord ROIs were highlighted using the 3D ROI drawing tool. (c) The spine ROI was then removed, leaving only the ROIs for the lumbar and thoracic/cervical spinal cords. (d) Brain ROIs of interest were obtained using a 3D mouse brain atlas: medulla (yellow), cerebellum (light blue), midbrain (dark green), pons (light pink), cortex (gray), thalamus (red), hypothalamus (light purple), hippocampus (dark purple), striatum (magenta), pallidum (peach), and olfactory bulbs (maroon). After fitting the 3D mouse brain atlas ROI to the CT, the amount of activity in each region was obtained in nCi/cm^3^ and then converted to %ID/g. (e) ROI of the right hemisphere was used for correlation with Western blot data, which was performed using only the right brain hemisphere for each mouse. (DOCX 749 kb)

Additional file 2: Dynamic PET imaging of [^18^F]FSPG uptake in the spinal cords (a) and brains (b). Time-activity curves represent average counts from a dynamic 90-min scan in EAE and naïve mice. Data are mean ± SEM (*n* = 3–7 animals per group). (DOCX 119 kb)

Additional file 3: [^18^F]FSPG and [^18^F]FDG ex vivo autoradiography of EAE versus naïve mice**.** Representative autoradiography images of whole spinal cords and sagittal brain sections from EAE (score 3.0–3.5) versus naïve mice collected ~ 110 min after injection of (a) [^18^F]FSPG and (b) [^18^F]FDG. The same brain sections were stained with Nissl and overlayed with the corresponding autoradiographic images. Cb—cerebellum, Ctx—cortex, HC—hippocampus, Med—medulla, Ob—olfactory bulb, Thal—thalamus. Dotted lines highlight region containing the optic chiasm. (DOC 184 kb)

Additional file 4: Adjacent coronal brain PET/CT images of a representative EAE mouse. Images of EAE mouse (score 1.5) were acquired ~ 75 min after injection of [^18^F]FSPG. Images are arranged beginning with caudal slices through to more rostral regions of the brain. Dotted lines highlight region of [^18^F]FSPG accumulation in meninges and/or in possible meningeal vessel(s) or subarachnoid space. (DOCX 2608 kb)

Additional file 5: Ex vivo biodistribution of [^18^F]FSPG in EAE versus control mice (a) with magnified views of the brain (b) and spinal cord (c) data (*n* = 8–10, mean %ID/g ± SD). Data was collected 110 min after injection of radiotracer and mean score of EAE mice was 2.4 ± 0.8. Significance determined Mann-Whitney test with ****p < 0.0001, ***p 0.0001–0.001, **p 0.001–0.01, *p 0.01–0.05. (DOCX 128 kb)

Additional file 6: Complete raw/unedited images of Western blots shown in Fig. 4a. Blot after incubation with anti-xCT antibody (a) the same blot having been re-probed with loading controls anti-actin and anti-Na,K-ATPase antibody (b). (DOCX 10919 kb)

Additional file 7: xCT protein levels in CNS tissues do not differ between control and naïve mice. SC = spinal cord. Data are represented as mean xCT/Na,K-ATPase signal ± SD (*n* = 3–5). Significance determined by Mann-Whitney test with NS = not significant. (DOCX 65 kb)

## Abbreviations

[^18^F]FDG: ^18^F-Fluorodeoxyglucose
[^18^F]FSPG: (4S)-4-(3-[^18^F]Fluoropropyl)-l-glutamate
BCA: Bicinchoninic acid
Bl: Bladder
Br: Brain
Cb: Cerebellum
CFA: Complete Freund’s adjuvant
CNS: Central nervous system
CT: Computed tomography
Ctrl: Control
Ctx: Cortex
EAE: Experimental autoimmune encephalomyelitis
ECL: Enhanced chemiluminescence
FBS: Fetal bovine serum
H: Haderian glands
HBSS: Hank’s buffered salt solution
HC: Hippocampus
I: Immunization site
i.p.: Intraperitoneal
i.v.: Intravenous
ID: Injected dose
K: Kidneys
LPS: Lipopolysaccharide
MAP: Maximum a posteriori
Mb: Midbrain
Med: Medulla
MOG: Myelin oligodendrocyte glycoprotein
MS: multiple sclerosis
NS: Not significant
O.C.T.: Optimal cutting temperature
Ob: Olfactory bulb
p.i.: Post injection
PBS: Phosphate buffered saline
PET: Positron emission tomography
PMBC: Peripheral mononuclear blood cell
ROI: Region of interest
ROS: Reactive oxygen species
RPMI medium: Roswell Park Memorial Institute medium
S: Salivary glands
SC: Spinal cord
SD: Standard deviation
Thal: Thalamus
xc-: Cystine/glutamate antiporter
xCT: Light chain of xc-
