# Supplementary material for: [18F]FSPG-PET reveals increased cystine/glutamate antiporter (xc-) activity in a mouse model of multiple sclerosis
Source: J Neuroinflammation. 2018 Feb 22;15:55. doi: 10.1186/s12974-018-1080-1 (PMC5822551; doi:10.1186/s12974-018-1080-1)
Supplement: Supplementary file 1 — Methods for defining regions of interest (ROIs) in CNS using VivoQuant image analysis software. (a) Spine/vertebrae (blue) was segmented out using Otsu Thresholding. (b) The spine ROI was made immutable, and the thoracic/cervical (orange) and lumbar (yellow) spinal cord ROIs were highlighted using the 3D ROI drawing tool. (c) The spine ROI was then removed, leaving only the ROIs for the lumbar and thoracic/cervical spinal cords. (d) Brain ROIs of interest were obtained using a 3D mouse brain atlas: medulla (yellow), cerebellum (light blue), midbrain (dark green), pons (light pink), cortex (gray), thalamus (red), hypothalamus (light purple), hippocampus (dark purple), striatum (magenta), pallidum (peach), and olfactory bulbs (maroon). After fitting the 3D mouse brain atlas ROI to the CT, the amount of activity in each region was obtained in nCi/cm3 and then converted to %ID/g. (e) ROI of the right hemisphere was used for correlation with Western blot data, which was performed using only the right brain hemisphere for each mouse. (DOCX 749 kb) [file 12974_2018_1080_MOESM1_ESM.docx]

**Additional File 1. Methods for defining regions of interest (ROIs) in CNS using VivoQuant image analysis software.** **(a)** Spine/vertebrae (blue) was segmented out using Otsu Thresholding. **(b)** The spine ROI was made immutable, and the thoracic/cervical (orange) and lumbar (yellow) spinal cord ROI’s were highlighted using the 3D ROI drawing tool. **(c)** The spine ROI was then removed, leaving only the ROIs for the lumbar and thoracic/cervical spinal cord. **(d)** Brain ROIs of interest were obtained using a 3D mouse brain atlas: medulla (yellow), cerebellum (light blue), midbrain (dark green), pons (light pink), cortex (grey), thalamus (red), hypothalamus (light purple), hippocampus (dark purple), striatum (magenta), pallidum (peach), olfactory bulbs (maroon). After fitting the 3D mouse brain atlas ROI to the CT, the amount of activity in each region was obtained in nCi/cc and then converted to %ID/g. **(e)** ROI of the right hemisphere was used for correlation with western blot data, which was performed using only the right brain hemisphere for each mouse.
