# Supplementary material for: [18F]FSPG-PET reveals increased cystine/glutamate antiporter (xc-) activity in a mouse model of multiple sclerosis
Source: J Neuroinflammation. 2018 Feb 22;15:55. doi: 10.1186/s12974-018-1080-1 (PMC5822551; doi:10.1186/s12974-018-1080-1)
Supplement: Supplementary file 2 — Dynamic PET imaging of [18F]FSPG uptake in the spinal cords (a) and brains (b). Time-activity curves represent average counts from a dynamic 90-min scan in EAE and naïve mice. Data are mean ± SEM (n = 3–7 animals per group). (DOCX 119 kb) [file 12974_2018_1080_MOESM2_ESM.docx]

**Additional File 2.** **Dynamic PET imaging of [^18^F]FSPG uptake in spinal cords (a) and brains (b).** Time-activity curves represent average counts from a dynamic 90-minute scan in EAE and naïve mice. Data are mean ± SEM (*n* = 3–7 animals per group).
