# Supplementary figures and images for: [18F]FSPG-PET reveals increased cystine/glutamate antiporter (xc-) activity in a mouse model of multiple sclerosis
Source: J Neuroinflammation. 2018 Feb 22;15:55. doi: 10.1186/s12974-018-1080-1 (PMC5822551; doi:10.1186/s12974-018-1080-1)

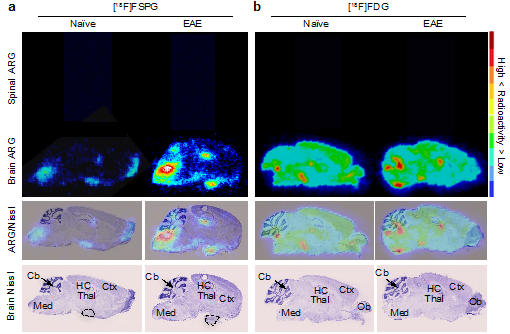

Supplement: Supplementary file 3 — [18F]FSPG and [18F]FDG ex vivo autoradiography of EAE versus naïve mice. Representative autoradiography images of whole spinal cords and sagittal brain sections from EAE (score 3.0–3.5) versus naïve mice collected ~ 110 min after injection of (a) [18F]FSPG and (b) [18F]FDG. The same brain sections were stained with Nissl and overlayed with the corresponding autoradiographic images. Cb—cerebellum, Ctx—cortex, HC—hippocampus, Med—medulla, Ob—olfactory bulb, Thal—thalamus. Dotted lines highlight region containing the optic chiasm. (DOC 184 kb) [file 12974_2018_1080_MOESM3_ESM.doc]
