# Supplementary material for: [18F]FSPG-PET reveals increased cystine/glutamate antiporter (xc-) activity in a mouse model of multiple sclerosis
Source: J Neuroinflammation. 2018 Feb 22;15:55. doi: 10.1186/s12974-018-1080-1 (PMC5822551; doi:10.1186/s12974-018-1080-1)
Supplement: Supplementary file 4 — Adjacent coronal brain PET/CT images of a representative EAE mouse. Images of EAE mouse (score 1.5) were acquired ~ 75 min after injection of [18F]FSPG. Images are arranged beginning with caudal slices through to more rostral regions of the brain. Dotted lines highlight region of [18F]FSPG accumulation in meninges and/or in possible meningeal vessel(s) or subarachnoid space. (DOCX 2608 kb) [file 12974_2018_1080_MOESM4_ESM.docx]

**Additional File 4.** **Adjacent coronal brain PET/CT images of a representative EAE mouse.** Images of EAE mouse (score 1.5) were acquired ~75 minutes after injection of [^18^F]FSPG. Images are arranged beginning with caudal slices through to more rostral regions of the brain. Dotted lines highlight region of [^18^F]FSPG accumulation in meninges and/or in possible meningeal vessel(s) or subarachnoid space.
