# Supplementary material for: [18F]FSPG-PET reveals increased cystine/glutamate antiporter (xc-) activity in a mouse model of multiple sclerosis
Source: J Neuroinflammation. 2018 Feb 22;15:55. doi: 10.1186/s12974-018-1080-1 (PMC5822551; doi:10.1186/s12974-018-1080-1)
Supplement: Supplementary file 5 — Ex vivo biodistribution of [18F]FSPG in EAE versus control mice (a) with magnified views of the brain (b) and spinal cord (c) data (n = 8–10, mean %ID/g ± SD). Data was collected 110 min after injection of radiotracer and mean score of EAE mice was 2.4 ± 0.8. Significance determined Mann-Whitney test with ****p < 0.0001, ***p 0.0001–0.001, **p 0.001–0.01, *p 0.01–0.05. (DOCX 128 kb) [file 12974_2018_1080_MOESM5_ESM.docx]

**Additional File 5.** ***Ex vivo* biodistribution of [^18^F]FSPG in EAE versus control mice** **(a)** with magnified views of brain **(b)** and spinal cord **(c)** data (n=8-10, mean %ID/g ± SD). Data was collected 110 min after injection of radiotracer and mean score of EAE mice was 2.4±0.8. Significance determined Mann-Whitney test with *****p* <0.0001, ****p* 0.0001-0.001, ***p* 0.001-0.01, **p* 0.01-0.05
