# Supplementary material for: [18F]FSPG-PET reveals increased cystine/glutamate antiporter (xc-) activity in a mouse model of multiple sclerosis
Source: J Neuroinflammation. 2018 Feb 22;15:55. doi: 10.1186/s12974-018-1080-1 (PMC5822551; doi:10.1186/s12974-018-1080-1)
Supplement: Supplementary file 6 — Complete raw/unedited images of Western blots shown in Fig. 4a. Blot after incubation with anti-xCT antibody (a) the same blot having been re-probed with loading controls anti-actin and anti-Na,K-ATPase antibody (b). (DOCX 10919 kb) [file 12974_2018_1080_MOESM6_ESM.docx]

**Additional File 6.** **Complete raw/unedited images of Western blots shown in Figure 4A**. Blot after incubation with anti-xCT antibody **(a)** the same blot having been re-probed with loading controls anti-actin and anti-Na,K-ATPase antibody **(b)**.
