# Supplementary material for: [18F]FSPG-PET reveals increased cystine/glutamate antiporter (xc-) activity in a mouse model of multiple sclerosis
Source: J Neuroinflammation. 2018 Feb 22;15:55. doi: 10.1186/s12974-018-1080-1 (PMC5822551; doi:10.1186/s12974-018-1080-1)
Supplement: Supplementary file 7 — xCT protein levels in CNS tissues do not differ between control and naïve mice. SC = spinal cord. Data are represented as mean xCT/Na,K-ATPase signal ± SD (n = 3–5). Significance determined by Mann-Whitney test with NS = not significant. (DOCX 65 kb) [file 12974_2018_1080_MOESM7_ESM.docx]

**Additional File 7.** **xCT protein levels in CNS tissues do not differ between control and naïve mice**. SC = spinal cord. Data are represented as mean xCT/Na,K-ATPase signal ± SD (n = 3-5). Significance determined by Mann-Whitney test with NS = not significant.
